# Response to antiviral therapy for chronic hepatitis C and risk of hepatocellular carcinoma occurrence in Japan: a systematic review and meta-analysis of observational studies

**DOI:** 10.1038/s41598-023-30467-5

**Published:** 2023-03-01

**Authors:** Yoko Yamagiwa, Keitaro Tanaka, Keitaro Matsuo, Keiko Wada, Yingsong Lin, Yumi Sugawara, Tetsuya Mizoue, Norie Sawada, Hidemi Takimoto, Hidemi Ito, Tetsuhisa Kitamura, Ritsu Sakata, Takashi Kimura, Shiori Tanaka, Manami Inoue, for the Research Group for the Development and Evaluation of Cancer Prevention Strategies in Japan, Sarah Krull Abe, Shuhei Nomura

**Affiliations:** 1 Division of Prevention, National Cancer Center Institute for Cancer Control, 5-1-1 Tsukiji, Chuo-ku, Tokyo, 104-0045 Japan; 2 Clinical Research Centers for Medicine, International University of Health and Welfare, Tokyo, Japan; 3 Department of Preventive Medicine, Faculty of Medicine, Saga University, Saga, Japan; 4 Division of Cancer Epidemiology and Prevention, Aichi Cancer Center Research Institute, Nagoya, Japan; 5 Department of Epidemiology and Preventive Medicine, Gifu University Graduate School of Medicine, Gifu, Japan; 6 Department of Public Health, Aichi Medical University School of Medicine, Aichi, Japan; 7 Division of Epidemiology, Department of Public Health and Forensic Medicine, Tohoku University Graduate School of Medicine, Sendai, Japan; 8 Department of Epidemiology and Prevention, Center for Clinical Sciences, National Center for Global Health and Medicine, Tokyo, Japan; 9 Division of Cohort Research, National Cancer Center Institute for Cancer Control, Tokyo, Japan; 10 Department of Nutritional Epidemiology, National Institute of Health and Nutrition, National Institute of Biomedical Innovation, Health and Nutrition, Tokyo, Japan; 11 Division of Cancer Information and Control, Department of Preventive Medicine, Aichi Cancer Center Research Institute, Nagoya, Japan; 12 Department of Social Medicine, Osaka University Graduate School of Medicine, Osaka, Japan; 13 Department of Epidemiology, Radiation Effects Research Foundation, Hiroshima, Japan; 14 Department of Public Health, Hokkaido University Graduate School of Medicine, Sapporo, Japan; 15 The University of Tokyo, Tokyo, Japan

**Keywords:** Gastroenterology, Oncology, Risk factors

## Abstract

In Japan, hepatocellular carcinoma (HCC) is a leading cause of cancer mortality and hepatitis C virus infection is a major cause of HCC. We conducted a systematic review and meta-analysis of published studies evaluating patient response to antiviral therapy for chronic hepatitis C on the risk of HCC occurrence in Japan. Articles were searched using terms determined a priori through PubMed, screened by title and abstract, and selected by full-text assessment according to criteria determined a priori, including HCC occurrence in response to interferon (IFN)-based or IFN-free therapy, Japanese study, and 2 or more years of follow-up. We excluded studies on HCC recurrence. We calculated the pooled estimate of the crude incidence rate ratio with data from the selected studies using the person-years method with Poisson regression model and pooled estimate of the hazard ratio adjusted for potential confounders reported by the studies using a random effects model. A total of 26 studies were identified, all of which examined only IFN-based therapy as a result of the selection process. The pooled estimate (95% confidence interval [CI]) of 25 studies was 0.37 (0.33–0.43) for sustained virologic response (SVR) and 1.70 (1.61–1.80) for non-SVR for the HCC incidence rate per 100 person-years, and 0.22 (0.19–0.26) for the incidence rate ratio (SVR vs. non-SVR). The pooled estimate of the hazard ratio (95% CI) of HCC incidence adjusted for potential confounders of 8 studies was 0.25 (0.19–0.34). SVR to interferon therapy for chronic hepatitis C reduces the risk of HCC occurrence.

## Introduction

Based on sufficient evidence for hepatocellular carcinoma (HCC) and non-Hodgkin lymphoma and a positive association with cholangiocarcinoma, the International Agency for Research on Cancer has stated that chronic infection with hepatitis C virus (HCV) is carcinogenic to humans^1^. Through adoption of the health-related goal to combat hepatitis in the Sustainable Development Goals in 2015^2^, the World Health Organization has further strengthened its global hepatitis program for prevention, screening, and antiviral therapy to eliminate viral hepatitis (hepatitis B and hepatitis C) by 2030 as a public health threat^3^. Globally, an estimated 58 million people had chronic HCV infection in 2021^4^ and HCV infection caused 140,000 cancer cases including HCC in 2018^5^. According to the WHO, improvements have been made to various policies for hepatitis C infection in Asian countries^6–13^. In Japan, chronic hepatitis C has been the leading cause of HCC, accounting for 60% of HCC incidence in 2015^14^. Further, HCC was the fifth leading cause of cancer mortality in 2019, and prognosis has not sufficiently improved, with the 5-year relative survival rate of liver cancer reported to be 35.8% in the population-based cancer registry (diagnosed in 2009–2011) in Japan^15^. According to molecular clock analysis using long-term serial samples of HCV in Japan, HCV infection was started around 1882, widely disseminated from the 1930’s, and decreased from 1995^16^. These data are supported by historical episodes: HCV infection is thought to have been expanded by therapy for Schistosoma japonicum from 1923, stimulant use around the second world war and contaminated blood transfusion/products from the 1950’s, and decreased by blood screening with HCV antibody from 1990 after the discovery of HCV in 1989 and the use of disposable medical supplies^16,17^. Epidemiological studies have shown a decreasing trend in the proportion of HCV carriers by birth year^18^. In 2015, an estimated 0.9–1.3 million people had chronic HCV infection^19^. Regarding antiviral therapy for chronic hepatitis C^20^, interferon (IFN) drugs were approved in 1992 simultaneously with the introduction of HCV screening for patients. The sustained virologic response (SVR) rate of antiviral therapy for genotype 1 of HCV has gradually improved over recent decades: it began at 5% with IFN monotherapy, increased to 30% with IFN combined with ribavirin from 2001, to 40–50% with pegylated IFN (Peg-IFN) combined with ribavirin from 2004, to over 70% with further addition of direct-acting antivirals from 2011, and then to over 95% with IFN-free therapy consisting of direct-acting antivirals from 2014.

A previous meta-analysis that included studies from Japan reported that a SVR to IFN-based therapy for chronic hepatitis C is an indicator of the risk of HCC occurrence^21^. The effectiveness of IFN-based therapy is determined by host and viral factors, the distributions of which are specific to various racial and regional characteristics*,* such as interleukin-28B polymorphism^22–24^ and HCV genotype^25,26^, respectively. While IFN-free therapy has the potential to overcome both determinants of effectiveness, studies on the association between direct acting antivirals and HCC incidence are sparse^27–29^.

Here, we conducted a systematic review and meta-analysis of the response to antiviral therapy for chronic hepatitis C and risk of HCC occurrence in the Japanese population. This work is part of a series of systematic reviews that summarize evidence available from cancer epidemiology and prevention research exclusively on Japanese subjects.

## Methods

### Study selection

Studies were identified through PubMed as of April 5, 2022, using the following search terms determined a priori: (interferon or daclatasvir or asunaprevir or sofosbuvir or ledipasvir or ombitasvir or paritaprevir or ritonavir or elbasvir or grazoprevir or beclabuvir or glecaprevir or pibrentasvir or velpatasvir or direct acting antiviral) AND (hepatitis c) AND (hepatocellular carcinoma or liver cancer or liver neoplasm) AND (Japan or Japanese). Two investigators (YY and KT) independently screened all article titles and abstracts, and conducted full-text assessment to determine eligibility, according to criteria determined a priori. Inclusion criteria were (1) studies on Japanese populations, (2) examination of HCC incidence related to SVR and non-SVR and/or non-IFN treatment as chronic hepatitis C therapy, and (3) follow-up duration of 2 or more years. The exclusion criteria were (1) examination of HCC recurrence, (2) examination of continuing therapy for chronic hepatitis C, (3) studies including participants with liver transplantation, (4) studies including participants with co-infection by human immunodeficiency virus, and (5) studies with less than 20 total participants or with less than 10 participants with SVR or non-SVR. For overlapping study populations identified by study period and institute, studies that included a more comprehensive population and more complete data were selected. Inconsistencies in study selection between the reviewers were solved by discussion.

### Data extraction and assessment of risk of bias

Data were extracted by YY using a spreadsheet developed a priori that included HCC cases with SVR/non-SVR, patients with SVR/non-SVR, follow-up period, hazard ratio (HR) adjusted for covariates, age, sex, advanced fibrosis, HCV genotype, and methods for HCC diagnosis. The data were checked by KT. Risk of bias in the selected studies was assessed by YY using the Newcastle Ottawa Scale for cohort studies, which consists of three items (selection, compatibility, and outcome) with eight sub-items^30^, and checked by KT. A “good” quality score required 3 or 4 stars in selection, 1 or 2 stars in comparability, and 2 or 3 stars in outcome. A “fair” quality score required 2 stars in selection, 1 or 2 stars in comparability, and 2 or 3 stars in outcome. A “poor” quality score reflected 0 or 1 star in selection, or 0 stars in comparability, or 0 or 1 star in outcome.

### Data synthesis and analysis

The crude incidence rate and incidence rate ratio for each study were calculated using the person-years method assuming a Poisson distribution of the observed number of HCC cases with SVR/non-SVR during the follow-up period. Pooled estimates of both measures were derived using a Poisson regression model with a random intercept and a random coefficient for IFN response (SVR vs. non-SVR) according to each study. A pooled estimate of the HR adjusted for potential confounders reported in the studies was calculated using a random effects model with restricted maximum likelihood. Subgroup analysis and sensitivity analysis were conducted on studies that did or did not include/report data on patients with liver cirrhosis, HBsAg, and Peg-IFN use. Heterogeneity among studies was assessed using *Q* statistics and *I*^*2*^ statistics. Publication bias was tested using funnel plots and Egger’s test. All analyses were performed with Stata 17.0 (Stata Corp LLC, College Station, TX). This systematic review was not registered.

## Results

### Study selection

**Figure 1:**
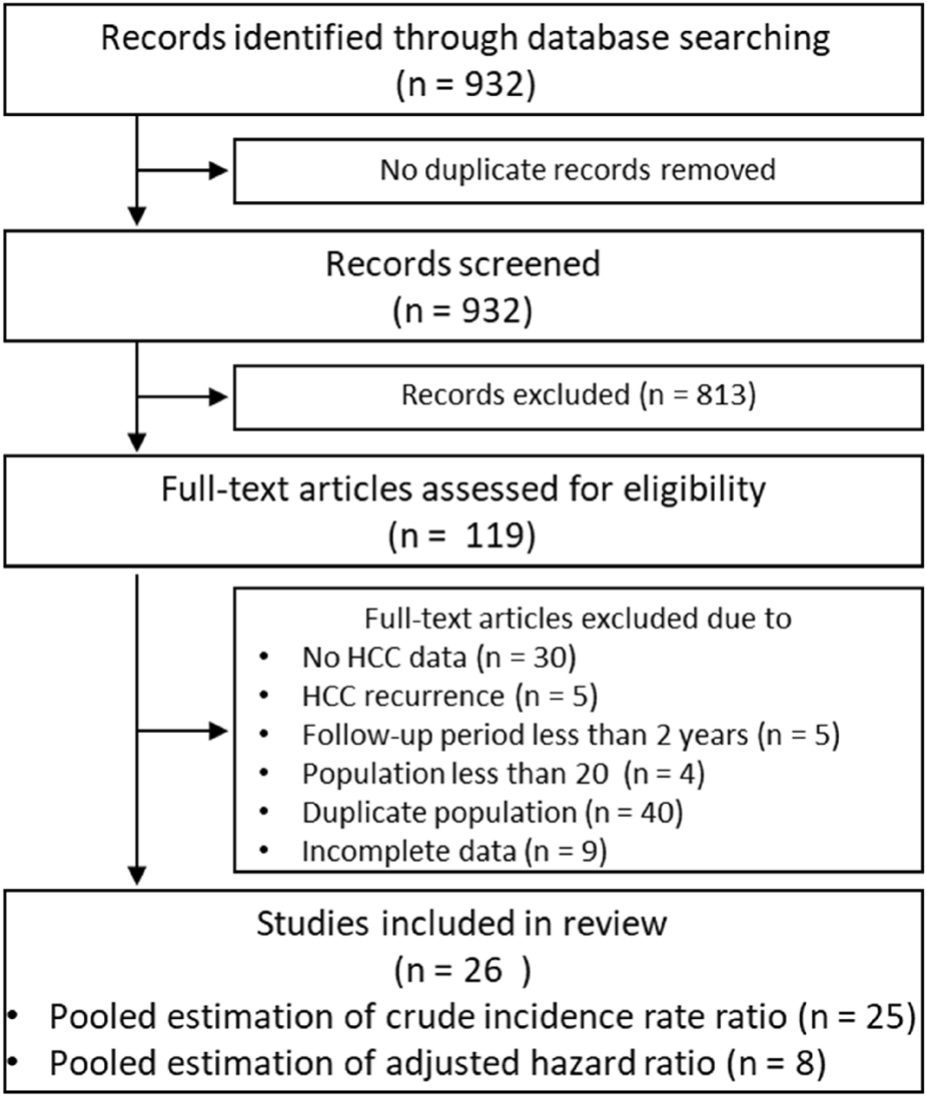
Flow diagram of study search and selection process. *HCC* hepatocellular carcinoma.

A total of 932 articles were identified through PubMed using search terms determined a priori on antiviral therapy for chronic hepatitis C (Fig. 1). Of these, 119 articles were selected by screening titles and abstracts. Full-text assessment according to the inclusion and exclusion criteria led to the exclusion of 93 studies due to unavailable data on HCC (n = 30), studies on HCC recurrence (n = 5), follow-up period less than 2 years (n = 5), population less than 20 (n = 4), duplicate population (n = 40), and incomplete data, including the number of HCC occurrences among those with SVR/non-SVR, patients with SVR/non-SVR, and follow-up period, or adjusted hazard ratio (n = 9). As a result, 26 studies on only IFN-based therapy, but not IFN-free therapy, were selected.

### Study characteristics and risk of bias assessment

**Figure 2:**
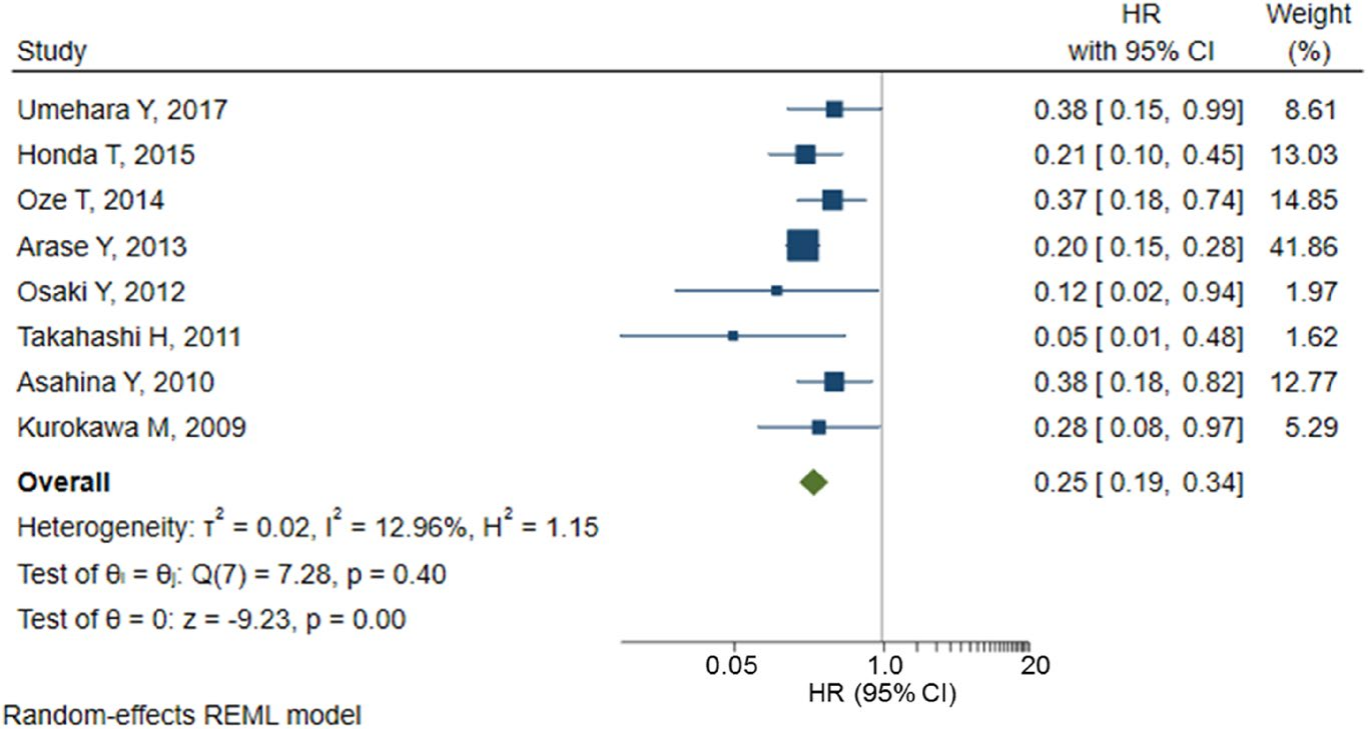
Forrest plot of the pooled estimate of the hazard ratio of HCC incidence adjusted for potential covariates in patients treated with antiviral therapy (SVR *vs.* non-SVR) in 8 studies. *HCC* hepatocellular carcinoma, *SVR* sustained virologic response, *HR* hazard ratio, *CI* confidence interval.

**Figure 3:**
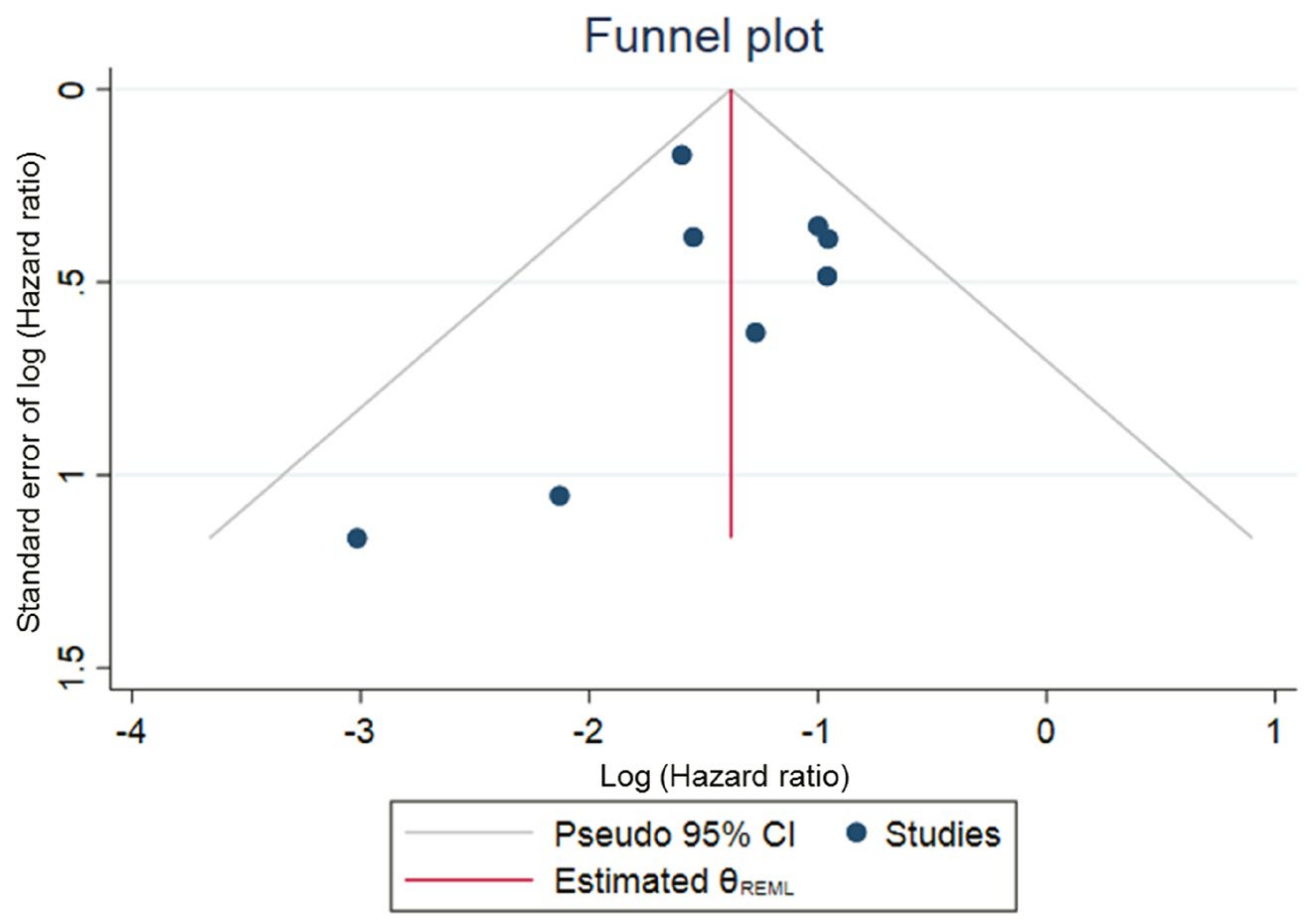
Funnel plot evaluating the publication bias of 8 studies used to determine the pooled estimate of the hazard ratio of HCC incidence adjusted for potential covariates in patients treated with antiviral therapy (SVR vs*.* non-SVR). *HCC* hepatocellular carcinoma, *SVR* sustained virologic response, *CI* confidence interval.

The characteristics of individual studies are summarized in Table 1. Year of publication, sample size, average follow-up duration and average age ranged from 1997 to 2017, 118 to 4302, 2.4 years to 11.8 years, and 47.2 years to 60 years, respectively. SVR was defined as undetectable HCV RNA at 24 weeks after the end of treatment in all studies, and the SVR rate ranged from 19.6% to 55.3%. In our risk of bias assessment using the Newcastle–Ottawa Scale for individual studies (Table 2), the exposure and outcome were reported based on medical records in most studies as they were hospital-based studies. The mean follow-up period was 5 or more years in 11 studies. The definition of “follow-up period” was inconsistent or unreported. The number lost to follow-up was reported in 6 of 26 studies. The overall study quality was regarded as “good” in 11 studies and “poor” in 15 studies. All eight studies that reported adjusted HRs had a “good” quality score. The magnitude of the association was consistent among studies (Table 3, Fig. 2). Publication bias cannot be ruled out due to asymmetry in the funnel plot (Fig. 3). No effects were observed for small studies using Egger’s test (*P* = 0.54). Heterogeneity among studies was small (*Q* = 7.28, *P* = 0.40; *I*^2^ = 12.96%). Further investigation into possible publication bias using a contour-enhanced funnel plot suggested that there may be missing studies in the bottom right-hand side of the plot (Supplementary Fig. 1), where the direction of the effect would be the same as that of the smaller effect magnitude of small studies in the area of non-significance. However, as no effects were observed for small studies using Egger’s test and pooled estimates did not change when fixed-effect and random-effect estimates were compared [HR (95%CI): 0.25 (0.19–0.34) in random-effect model (REML); HR (95%CI): 0.24 (0.19–0.31) in fixed-effect model (inverse-variance)], asymmetry in the funnel plots might indicate the low impact of publication bias or be due to chance.

**Table 1:** Characteristics of studies included in the present systematic review on response to antiviral therapy for chronic hepatitis C and hepatocellular carcinoma occurrence.

Article	Study design			Study population										Cases					Relative risk	
Author, year/ref	Study design	Study period	Follow-up duration (years)	N	Age (years)	Men (%)	Advanced fibrosis^a^ (%)	HCV GT1 (%)	IFN	SVR rate (%)	No of SVR	No of non-SVR	No of non-IFN	SVR	Non-SVR	Non-IFN	Diagnosis method for HCC		Adjusted HR (95%CI)	Covariates
Umehara Y 2017 ^46^	Retrospective hospital-based single	2004–2014	N/R	415	58	46.0	25.2	67.5	N/R	N/R	N/R	N/R	N/A	N/R	N/R	N/A	US/CT/MRI	Non-SVR	Ref	Age, fibrosis stage, Plt, Alb, ALT, AFP
																		SVR	0.38 (0.15–0.99)	
Tada T 2016 ^47^	Retrospective hospital-based single	1994–2014	11.8	2743	60	52.6	APRI 0.69	53.8	N/R	55.3	587	475	1681	31	131	412	US/CT/MRI	Non-IFN	Ref	Age, sex, AST, ALT,
																		SVR	0.28 (0.16–0.45)	Alb, T-bil, PT, Plt,
Takeuchi Y 2015 ^48^	Retrospective hospital-based multi	2005–2011	5.4	1255	59	54.0	N/R	67.4	Peg	53.0	665	590	N/A	20	69	N/A	US/CT/MRI/Angiography/Biopsy	N/R	N/R	AFP, HCV GT
Honda T 2015 ^49^	Retrospective hospital-based multi	2004–2010	3.92	1280	54.2	52.2	13.9	67.7	Peg	54.8	701	579	N/A	9	41	N/A	US/CT/MRI/Angiography/Biopsy	SVR	Ref	Age, sex, advanced fibrosis
																		Non-SVR	4.69 (2.21–9.94)	
Oze T 2014 ^50^	Prospective hospital-based multi	2002–2008	3.08	2600	56.4	47.9	15.8	77.1	Peg	54.8	1425	1175	N/A	23	81	N/A	US/CT/MRI/Angiography/Biopsy	Non-SVR	Ref	Age, sex, fibrosis score, Plt, T-bil, Alb, ALT, AFP
																		SVR	0.37 (0.18–0.74)	
Ogawa E 2013 ^51^	Prospective hospital-based multi	2004–2009	3.6	1013	58	49.2	14.8	70.1	Peg	55.0	557	456	N/A	13	34	N/A	US/CT/Biopsy	SVR	Ref	Age, sex, Plt, AFP, fibrosis
															TVR 13			TVR	1.50 (0.65–3.44)	
															NVR 21			NVR	3.72 (1.69–8.18)	
Arase Y 2013 ^44^	Retrospective hospital-based single	1990–2009	8.1	4302	52	58.8	10.1	63.2	N/R	44.2	1900	2402	N/A	44	349	N/A	US/CT/MRI/Biopsy	SVR	Ref	Age, sex, DM, alcohol intake, hepatic fibrosis
																		Non-SVR	4.93 (3.53–6.89)	
Osaki Y 2012 ^52^	Retrospective hospital-based single	2002–2010	4.1	382	59	50.3	N/R	59.9	Peg/others	48.4	185	197	N/A	1	22	N/A	US/CT/MRI	SVR	Ref	Age, ALT, AFP, Plt
																		Non-SVR	8.41 (1.07–66.30)	
Watanabe S 2011 ^53^	Retrospective hospital-based multi	2004–2007	4.25	1865	55.6	56.5	21.7	79	Peg	53.6	999	866	N/A	10	49	N/A	US/CT/Angiography/Biopsy	SVR	Ref	Age, sex, ALT, Plt, Alb
																		TVR	2.06 (0.71–5.96)	
																		NVR	2.99 (1.04–8.60)	
Takahashi H 2011 ^54^	Retrospective hospital-based single	2002–2007	4.33	203	55.4	53.2	23.2	74.9	Peg/others	43.8	89	114	N/A	1	12	N/A	US/CT	SVR	Ref	Age, sex, alcohol intake fibrosis, AFP, steatosis, OGTT
																		Non-SVR	20.4 (2.1–200.9)	
Asahina Y 2010 ^55^	Retrospective hospital-based multi	1992–2008	7.5	2166	55.4	49.9	25.2	65.6	Peg/others	33.6	686	1356	N/A	22	149	N/A	US/CT/MRI/Biopsy	SVR	Ref	Age, sex, fibrosis, steatosis, Tcho, FBS, AFP etc
																		Non-SVR	2.6 (1.2–5.5)	
Kurokawa M 2009 ^56^	Retrospective blood donation/ hospital-based	2002–2005	3.04	403	55.8	63.8	31.3	64.8	Others	34.5	139	264	N/A	4	21	N/A	US/CT/Angiography/Biopsy	SVR	Ref	Age, sex, fibrosis
																		Non-SVR	3.57 (1.04–12.36)	
Ikeda K 2007 ^57^	Prospective hospital-based multi	1995	9.5	576	59.6	57.3	Hepatitis	N/R	Others	23.7	53	171	352	1	34	59	US/CT	Non-IFN	Ref	Age, sex, alcohol intake, smoking status. HBcAb
																		SVR	0.15 (0.02–1.09)	
																		No response	1.84 (1.14–2.99)	
																		Relapse	0.50 (0.16–1.62)	
Kobayashi S 2007 ^58^	Retrospective hospital-based multi	1992–2003	5.5	1124	52	60.9	N/R	N/R	Others	33.2	373	751	N/A	13	61	N/A	US/CT/MRI/Angiography/Biopsy	N/R	N/R	N/A
Soga K 2005 ^59^	Retrospective hospital-based single	1992–1996	7.8	133	52.5	47.4	18.4	52.6	Others	32.0	33	70	30	0	5	7	US/CT/Angiography/Biopsy	N/R	N/R	N/A
Kim KI 2005 ^60^	Retrospective hospital-based single	N/R	SVR 2.68	118	N/R	N/R	14.4	N/R	N/R	26.3	31	87	N/A	1	8	N/A	US/CT/Biopsy	N/R	N/R	N/A
			PR 2.39																	
			NR 2.45																	
Moriyama M 2003 ^61^	Retrospective hospital-based single	1987–1998	6.46	654	48.9	59.5	N/R	53.2	Others	34.9	208	388	58	4	23	19	US/CT/Angiography/Biopsy	N/R	N/R	N/A
Ohata K 2003 ^62^	Retrospective hospital-based single	1980–1999	6.4	161	53	65.8	54.7	65.8	N/R	28.2	20	51	90	0	8	N/R	US/CT/Angiography	N/R	N/R	N/A
Tazawa J 2002 ^63^	Retrospective hospital-based single	1987–1995	5.49	279	49.4	68.1	23.7	48.7	Others	29.7	55	130	94	0	8	5	US/CT/Angiography/Biopsy	N/R	N/R	N/A
Yoneyama K 2002 ^45^	Retrospective hospital-based single	1984–1999	4.39	121	49.3	67.8	N/R	47.1	Others	43.8	53	68	N/A	1	9	N/A	US/CT/MRI/Biopsy	N/R	N/R	N/A
Hirashima N 2002 ^64^	Retrospective hospital-based single	1992–1994	4.9	153	51.7	68.6	30.7	74.5	Others	32.7	50	103	N/A	5	12	N/A	US/CT/Angiography/Biopsy	N/R	N/R	Age, sex, HCV GT, RNA, ALT, stage, grade
																			Effect of IFN therapy	
																			3.89 (0.53–3.34)	
Yoshida H 1999 ^65^	Retrospective hospital-based multi	1986–1998	4.3	2890	50.2	62.3	33.2	44.1	Others	33.5	789	1568	490	10	79	59	US/CT/MRI/Angiography/Biopsy	Non-IFN	Ref	Age, sex, fibrosis
																		SVR	0.20 (0.10–0.39)	
																		Non-SVR	0.61 (0.43–0.92)	
Horiike N 1998 ^66^	Retrospective hospital-based single	N/R	7.6	149	47.4	69.8	22.1	53	Others	37.5	33	55	61	0	1	9	N/R	N/R	N/R	N/A
Miyajima I 1998 ^67^	Retrospective hospital-based single	1991–1996	SVR2.3	213	47.2	63.8	28.6	66.2	Others	29.6	63	150	N/A	0	12	N/A	US/CT/Angiography/Biopsy	N/R	N/R	N/A
			Non-SVR2.5																	
Onodera H 1997 ^68^	Retrospective hospital-based single	1992–1996	SVR 129.5py	313	51.6	51.6	N/R	N/R	Others	34.1	59	114	140	0	4	3	US/CT/MRI/Angiography/Biopsy	N/R	N/R	N/A
			Non-SVR 246.25py																	
			Non-IFN 333.3py																	
Kuwana K 1997 ^69^	Retrospective hospital-based single	1988–1994	4.67	240	56.2	N/R	Excluding liver cirrhosis	N/R	Others	19.6	18	74	148	0	5	69	US/CT/MRI/angiography/biopsy	N/R	N/R	N/A

*AFP* alpha fetoprotein, *Alb* albumin, *ALT* alanine aminotransferase, *AST* aspartate aminotransferase, *CI* confidence interval, *CT* computed tomography, *DM* diabetes mellitus, *FBS* fasting blood sugar, *GT* genotype, *HCV* hepatitis C virus, *HR* hazard ratio, *IFN* interferon, *MRI* magnetic resonance imaging, *N or No* number, *N/R* not recorded, *N/A* not appreciable, *NVR* non-viral response, *OGTT* oral glucose tolerance test, *Peg* pegylated, *Plt* platelet, *PR* partial response, *PT* prothrombin time, *py* person-years, *Ref* reference, *RNA* ribonucleic acid, *SVR* sustained virologic response, *T-bil* total bilirubin, *TCho* total cholesterol, *US* ultrasound.

^a^Stage 3 or 4 liver fibrosis was determined by liver biopsy.

**Table 2:** Risk of bias assessment using the Newcastle–Ottawa Scale for 26 studies.

Article	Selection				Comparability		Outcome		
Author (year)	Representativeness of the exposed cohort^a^	Selection of the non-exposed cohort^b^	Ascertainment of exposure^c^	Outcome of interest was not present at start of study^d^	Adjusted for most important factor^e^	Adjusted for additional factors^f^	Assessment of outcome^g^	Follow-up long enough for outcomes to occur^h^	Adequacy of follow up of cohorts^i^
Umehara Y (2017)			●	●	●	●	●		
Tada T (2016)	○	●	●	●			●	●	
Takeuchi Y (2015)	●	●	●	●			●	●	
Honda T (2015)	●	●	●	●	●	●	●	○	
Oze T (2014)	●	●	●	●	●	●	●	○	
Ogawa E (2013)	●	●	●	●	●	●	●	○	
Arase Y (2013)	○	●	●	●	●	●	●	●	●
Osaki Y (2012)	○	●	●	●	●	●	●	○	
Watanabe S (2011)	●	●	●	●	●	●	●	○	
Takahashi H (2011)	○	●	●	●	●	●	●	○	
Asahina Y (2010)	●	●	●	●	●	●	●	●	
Kurokawa M (2009)	●	●	●	●	●	●	●	○	
Ikeda K (2007)	●	●	●	●		●	●	●	●
Kobayashi S (2006)	●	●	●	●			●	●	
Soga K (2005)	○	●	●	●			●	●	
Kim KI (2005)		●	●				●		
Moriyama M (2003)	○	●	●	●			●	●	
Ohata K (2003)	○	●	●	●			●	●	
Tazawa J (2002)	○	●	●	●			●	●	●
Yoneyama K (2002)	○	●	●				●	○	●
Hirashima N (2002)	○	●	●	●			●	○	
Yoshida H (1999)	●	●	●	●	●	●	●	○	●
Horiike N (1998)		●	●					●	
Miyajima I (1998)	○	●	●	●			●		
Onodera H (1997)	○	●	●				●		●
Kuwana K (1997)	○	●	●	●			●	○	

^a^Single (○) or multi-center (●) hospital-based study.

^b^Drawn from the same community as the exposed cohort.

^c^Based on medical records.

^d^Demonstrated.

^e^Adjusted for fibrosis.

^f^Adjusted for age and other factors.

^g^Based on medical records.

^h^Mean follow-up duration > 3 years (○) and > 5 years (●).

^i^Reported number lost to follow-up.

**Table 3:** Pooled estimates of the crude incidence rate (per 100 person-years) and incidence rate ratio of HCC in patients treated with antiviral therapy (SVR *vs.* non-SVR) calculated using data from 25 studies.

Author	Cases		100 person-years		Incidence rate (per 100 person-years)		Incidence rate ratio
	SVR	Non-SVR	SVR	Non-SVR	Pooled estimate (95% CI)		Pooled estimate (95% CI)
					SVR	Non-SVR	
Tada T	31	131	82.18	67.45	0.38 (0.26–0.56)	1.94 (1.62–2.31)	0.19 (0.13–0.29)
Takeuchi Y	20	69	35.91	31.86	0.56 (0.34–0.86)	2.17 (1.69–2.74)	0.26 (0.15–0.43)
Honda T	9	41	27.46	22.68	0.33 (0.15–0.62)	1.81 (1.30–2.45)	0.18 (0.08–0.38)
Oze T	23	81	47.50	39.17	0.48 (0.31–0.73)	2.07 (1.64–2.57)	0.23 (0.14–0.38)
Ogawa E	13	34	20.05	16.42	0.65 (0.35–1.11)	2.07 (1.43–2.89)	0.31 (0.15–0.61)
Arase Y	44	349	153.90	194.56	0.29 (0.21–0.38)	1.79 (1.61–1.99)	0.16 (0.11–0.22)
Osaki Y	1	22	7.59	8.08	0.13 (0.003–0.74)	2.72 (1.71–4.12)	0.05 (0.001–0.30)
Watanabe S	10	49	42.46	36.81	0.24 (0.11–0.43)	1.33 (0.99–1.76)	0.18 (0.08–0.35)
Takahashi H	1	12	3.86	4.94	0.26 (0.007–1.45)	2.43 (1.26–4.24)	0.11 (0.002–0.72)
Asahina Y	22	149	51.45	101.70	0.43 (0.27–0.65)	1.47 (1.24–1.72)	0.29 (0.18–0.46)
Kurokawa M	4	21	4.23	8.03	0.95 (0.26–2.42)	2.62 (1.62–4.00)	0.36 (0.09–1.07)
Ikeda K	1	34	5.04	16.25	0.20 (0.005–1.11)	2.09 (1.45–2.93)	0.10 (0.002–0.57)
Kobayashi S	13	61	20.52	41.31	0.63 (0.34–1.08)	1.48 (1.13–1.90)	0.43 (0.22–0.79)
Soga K	0	5	2.57	5.46	0 (0–1.43)	0.92 (0.30–2.14)	0 (0–2.32)
Kim KI	1	8	0.83	2.11	1.20 (0.03–6.71)	3.80 (1.64–7.49)	0.32 (0.007–2.36)
Moriyama M	4	23	13.44	25.06	0.30 (0.08–0.76)	0.92 (0.58–1.38)	0.32 (0.08–0.95)
Ohata K	0	8	1.28	3.26	0 (0–2.88)	2.45 (1.06–4.83)	0 (0–1.49)
Tazawa J	0	8	3.02	7.14	0 (0–1.22)	1.12 (0.48–2.21)	0 (0–1.39)
Yoneyama K	1	9	2.33	2.99	0.43 (0.01–2.39)	3.01 (1.38–5.72)	0.14 (0.003–1.03)
Hirashima N	5	12	2.47	5.08	2.03 (0.66–4.73)	2.36 (1.22–4.13)	0.86 (0.24–2.62)
Yoshida H	10	79	33.93	67.42	0.30 (0.14–0.54)	1.17 (0.93–1.46)	0.25 (0.12–0.49)
Horiike N	0	1	2.11	3.52	0 (0–1.75)	0.28 (0.01–1.58)	0 (0–65.00)
Miyajima I	0	12	1.45	3.75	0 (0–2.55)	3.20 (1.65–5.59)	0 (0–0.93)
Onodera H	0	4	2.46	3.33	0 (0–1.5.00)	1.20 (0.33–3.07)	0 (0–2.05)
Kuwana K	0	5	0.84	3.46	0 (0–4.38)	1.45 (0.47–3.37)	0 (0–4.48)
Pooled estimate					0.38 (0.32–0.46)	1.75 (1.54–1.99)	0.22 (0.18–0.26)
Cumulative incidence (%)				1 year	0.38 (0.32–0.46)	1.74 (1.53–1.98)	
				5 years	1.90 (1.58–2.29)	8.39 (7.41–9.49)	
				10 years	3.76 (3.13–4.52)	16.07 (14.27–18.08)	

*HCC* hepatocellular carcinoma, *SVR* sustained virologic response, *CI* confidence interval.

### Pooled estimates of the crude incidence rate ratio

The crude incidence rate ratio was calculated using data from 25 studies (Table 3). HCC was not observed in patients with SVR in 7 studies. The pooled estimate (95% confidence interval [CI]) was 0.37 (0.33–0.43) in patients with SVR and 1.70 (1.61–1.80) in patients with non-SVR for the HCC incidence rate per 100 person-years, and 0.22 (0.19–0.26) for the incidence rate ratio (SVR *vs.* non-SVR). In subgroup analysis, pooled estimates of the incidence rate ratio did not differ by inclusion or exclusion of patients with HBsAg, liver cirrhosis, or Peg-IFN use (Table 4).

**Table 4:** Subgroup analyses of pooled estimates of the crude incidence rate (per 100 person-years) and incidence rate ratio of HCC in patients treated with antiviral therapy (SVR vs. non-SVR) related to liver cirrhosis, HBsAg positivity, and Peg-IFN use.

Studies	Incidence rate (per 100 person-years)		Incidence rate ratio
	Pooled estimate (95% CI)		Pooled estimate (95% CI)
	SVR	Non-SVR	
Liver cirrhosis			
Included (n = 19)	0.38 (0.33–0.44)	1.71 (1.61–1.81)	0.22 (0.19–0.26)
Excluded (n = 6)	0.14 (0.04–0.56)	1.61 (1.25–2.07)	0.09 (0.02–0.35)
HBsAg positive			
Included or unreported (n = 9)	0.38 (0.28–0.53)	1.80 (1.56–2.09)	0.21 (0.15–0.30)
Excluded (n = 16)	0.37 (0.32–0.43)	1.68 (1.58–1.79)	0.22 (0.19–0.26)
Peg-IFN			
Other or unreported (n = 17)	0.34 (0.29–0.41)	1.67 (1.56–1.80)	0.21 (0.17–0.25)
Included (n = 8)	0.42 (0.34–0.51)	1.75 (1.59–1.91)	0.24 (0.19–0.30)

*HCC* hepatocellular carcinoma, *SVR* sustained virologic response, *Peg-IFN* pegylated interferon, *CI* confidence interval.

### Pooled estimate of adjusted hazard ratio

**Figure 4:**
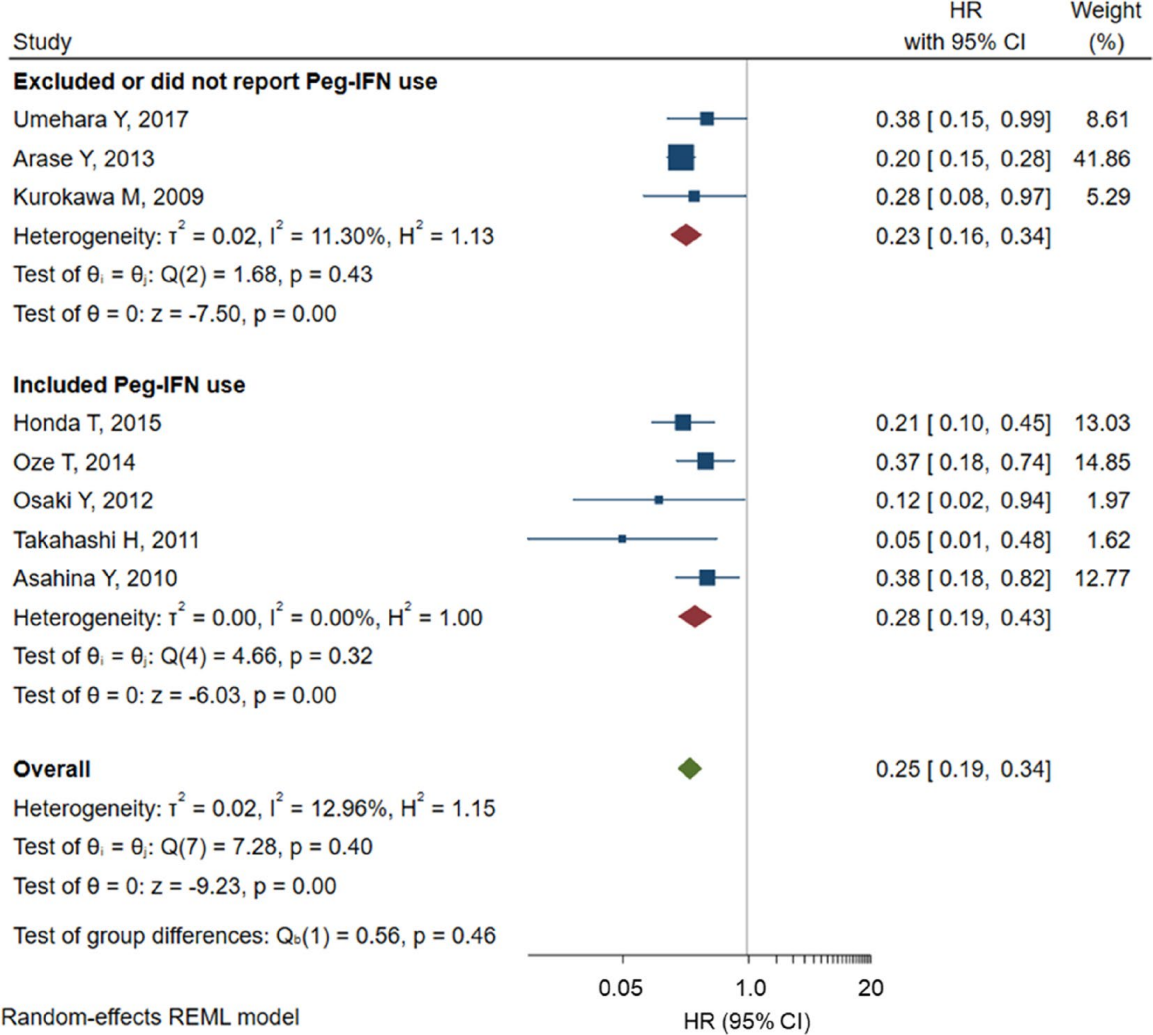
Forrest plot of the pooled estimate of the hazard ratio of HCC incidence adjusted for potential covariates in patients treated with antiviral therapy (SVR vs*.* non-SVR) by studies that did and did not include Peg-IFN use. *HCC* hepatocellular carcinoma, *SVR* sustained virologic response, *Peg-IFN* pegylated interferon, *HR* hazard ratio, *CI* confidence interval.

Eight studies reported the HR (SVR *vs.* non-SVR) adjusted for covariates of liver fibrosis including fibrosis stage and platelet count. The pooled estimate of the adjusted HR (95% CI) of HCC incidence was 0.25 (0.19–0.34) (Fig. 2). In subgroup analysis, pooled estimates of the adjusted HR did not differ by the inclusion or exclusion of patients with Peg-IFN use (Fig. 4). In sensitivity analysis, the pooled estimate of the adjusted HR of studies that did and did not include HBsAg-positive patients (n = 7; pooled estimate of adjusted HR = 0.25, 95% CI: 0.19–0.34) did not differ. Patients with liver cirrhosis were included in all 8 studies. Pooled estimates did not change in sensitivity analyses that excluded poor quality studies (incidence rate ratio (95% CI): 0.21 (0.17–0.25) in fair studies (n = 11); HR (95% CI): 0.24 (0.18–0.33) in fair studies (n = 7)). Likewise, pooled estimates did not change in subgroup analyses on study design, study period, follow-up period, sample size, and age (Supplementary Table 1 and 2).

## Discussion

We conducted a systematic review of studies that examined the association between response to antiviral therapy for chronic hepatitis C and risk of HCC occurrence in a Japanese population. Based on pooled estimates of both the crude incidence rate ratio calculated using data from individual studies and the HR adjusted for potential confounders reported by the studies, we concluded that SVR to IFN-based therapy for chronic hepatitis C reduces the risk of HCC occurrence. In subgroup analysis and sensitivity analysis related to HBsAg-positivity, liver cirrhosis, and Peg-IFN use, pooled estimates of each effect size did not change.

A previous meta-analysis of observational studies in Asia, including Japan, Europe and North America reported an effect size of similar magnitude to that reported in the present study, irrespective of whether patients had advanced liver fibrosis: SVR to IFN-based therapy reduced the risk of HCC occurrence overall (pooled HR = 0.24, 95% CI: 0.18–0.31) and in advanced liver fibrosis (pooled HR = 0.23, 95% CI: 0.16–0.35)^21^.

Eradication of HCV by IFN is thought to reduce HCC occurrence by improving hepatic inflammation, regression of hepatic fibrosis, and the antitumor effects of IFN including tumoricidal, antiproliferative, or immunomodulatory effects^31–33^. These actions are considered unique to IFN compared to IFN-free therapy. Although achievement of SVR to antiviral therapy reduces HCC occurrence, HCC risk can remain during follow-up in patients with SVR. Advanced fibrosis, older age, alcohol intake, and diabetes mellitus have been suggested to increase the risk of HCC^34^. Strategies to improve lifestyle factors along with surveillance for HCC occurrence in patients with SVR are still needed.

Due to the selection process, this study did not include articles on IFN-free therapy. Although the effect of SVR to IFN-free therapy on HCC occurrence was controversial in earlier studies due to the older age and more advanced fibrosis among patients receiving IFN-free therapy^35,36^, later studies suggested that SVR to IFN-free therapy did in fact reduce the risk of HCC^37^. In Japan, Kobayashi et al. reported a cumulative HCC incidence (3-/5-year) of 1.30/3.03% for IFN-free and 1.02/2.19% for IFN-based therapy during the follow-up period (median, 4.0 years and 7.3 years, respectively), with a log-rank test indicating no significant differences in either group^27^. Similarly, Nagata et al. demonstrated that the 3-year cumulative incidence of HCC occurrence in patients with SVR did not differ by therapy in propensity score-matched analysis (3.3% for IFN-based, 1.4% for IFN-free therapy; *P* = 0.49, log-rank test)^28^. Tahata et al. also reported that HCC occurrence in patients with SVR did not differ by therapy in propensity score-matched analysis (cumulative rates of HCC occurrence at 1 year and 2 years: 0.5% and 1.9% in the IFN-based group vs 1.1% and 3.0% in the IFN-free group, *P* = 0.489; adjusted HR (95% CI) vs IFN-based: 1.134 (0.367–3.498), *P* = 0.827)^38^.

Given that the effectiveness of IFN-based therapy is linked to host and viral factors, one strength of this study was that we focused our analysis specifically on the Japanese population. Among host factors such as age, sex, race, and liver fibrosis, a comparison of patient response to Peg-IFN therapy combined with ribavirin in those infected with HCV genotype 1 identified interleukin-28B polymorphisms as a contributing factor^22–24^. The SVR rate was 14% in patients with the unfavorable allele TG/GG at rs809917, and 50% in those with the favorable allele TT^39^. These polymorphisms are associated with spontaneous clearance of HCV, which is observed in around 30% of those with acute infection with HCV in Japan. The minor allele frequency of rs809917 or rs12979860 is race specific, and the frequency of the favorable allele is higher in Asians compared with Caucasians and African-Americans^40–42^. In terms of viral factors, HCV genotype is related to response to antiviral therapy and the distribution of genotypes differs by region and route of transmission. Genotype 1 and poor response to IFN therapy were found in an estimated 65% of patients with chronic hepatitis C in 2015 in Japan^26^. Interestingly, evidence suggests that the distribution of genotypes has been altered with recent changes to the route of transmission, such as through intravenous drug abuse and tattoos, leading the prevalence of genotype 2b to be higher in patients born after 1970 regardless their place of birth in Japan^43^. The reported SVR rate to Peg-IFN therapy combined with ribavirin for genotype 2 is 80%^20^. Our findings on Japanese patients in this study suggest that eradication of HCV with antiviral therapy could be reducing HCC occurrence. Given the difficulty of comparing SVR and non-SVR to IFN-free therapy due to the high effectiveness of IFN-free therapy, further studies comparing SVR to IFN-free therapy and SVR to IFN are needed to evaluate the effects of SVR to IFN-free therapy on HCC occurrence.

Several limitations of this study should be noted. First, as most studies did not report the number of patients lost to follow-up, we could not rule out some risk of selection bias. Second, the definition of follow-up period was inconsistent or unreported among the selected studies; thus, HCC incidence may have been incorrectly evaluated in this study. Third, adjustment for confounders may have been insufficient: adjustment for alcohol consumption, smoking status, diabetes mellitus, and obesity was not necessarily conducted in each study. Furthermore, the effect size of HCC occurrence in patients with non-SVR to IFN therapy could have been overestimated, since older age and higher fibrosis stage are unfavorable factors for both response to IFN-based therapy and risk of HCC occurrence. Fourth, HCC cases with SVR were not observed and adjusted HRs were not available in earlier studies, possible because the effectiveness of antiviral therapy and technology used to diagnose HCC improved over the study period. Fifth, we used Pubmed only to search for reports during the selection process due to availability. Finally, we could not contact the study authors to clarify any unclear information because the studies were conducted a long time ago.

Future studies should examine the effects of SVR to IFN-free therapy on HCC occurrence by comparing SVR to IFN-free therapy and SVR to IFN. The findings of the present study suggest that eradication of HCV by IFN therapy may reduce HCC occurrence in Japanese patients. However, among the articles selected based on the eligible criteria in this study, only two articles reported outcomes other than HCC by response to antiviral therapy: unchanged incidence of malignancy other than HCC by response to IFN^44^ and decreased progression to LC in patients without LC before IFN therapy by SVR to IFN^45^. Outcomes other than HCC including overall-mortality, hepatic decompensation, and liver-related mortality by response to antiviral therapy are also important issues and warrant further study.

In conclusion, our systematic review on the association between response to antiviral therapy for chronic hepatitis C and HCC occurrence in a Japanese population suggests that eradication of HCV using antiviral therapy for chronic hepatitis C reduces HCC occurrence.

## Supplementary Information

Supplementary Information.

## Data Availability

The datasets generated during and/or analyzed during the current study are not publicly available due to the agreement of the Research Group for the Development and Evaluation of Cancer Prevention Strategies in Japan but are available from the corresponding author on reasonable request.
