# Author Correction: Response to antiviral therapy for chronic hepatitis C and risk of hepatocellular carcinoma occurrence in Japan: a systematic review and meta-analysis of observational studies

**DOI:** 10.1038/s41598-023-31052-6

**Published:** 2023-03-08

**Authors:** Yoko Yamagiwa, Keitaro Tanaka, Keitaro Matsuo, Keiko Wada, Yingsong Lin, Yumi Sugawara, Tetsuya Mizoue, Norie Sawada, Hidemi Takimoto, Hidemi Ito, Tetsuhisa Kitamura, Ritsu Sakata, Takashi Kimura, Shiori Tanaka, Manami Inoue, for the Research Group for the Development and Evaluation of Cancer Prevention Strategies in Japan

**Affiliations:** 1 Division of Prevention, National Cancer Center Institute for Cancer Control, 5-1-1 Tsukiji, Chuo-ku, Tokyo, 104-0045 Japan; 2 Clinical Research Centers for Medicine, International University of Health and Welfare, Tokyo, Japan; 3 Department of Preventive Medicine, Faculty of Medicine, Saga University, Saga, Japan; 4 Division of Cancer Epidemiology and Prevention, Aichi Cancer Center Research Institute, Nagoya, Japan; 5 Department of Epidemiology and Preventive Medicine, Gifu University Graduate School of Medicine, Gifu, Japan; 6 Department of Public Health, Aichi Medical University School of Medicine, Aichi, Japan; 7 Division of Epidemiology, Department of Public Health and Forensic Medicine, Tohoku University Graduate School of Medicine, Sendai, Japan; 8 Department of Epidemiology and Prevention, Center for Clinical Sciences, National Center for Global Health and Medicine, Tokyo, Japan; 9 Division of Cohort Research, National Cancer Center Institute for Cancer Control, Tokyo, Japan; 10 Department of Nutritional Epidemiology, National Institute of Health and Nutrition, National Institute of Biomedical Innovation, Health and Nutrition, Tokyo, Japan; 11 Division of Cancer Information and Control, Department of Preventive Medicine, Aichi Cancer Center Research Institute, Nagoya, Japan; 12 Department of Social Medicine, Osaka University Graduate School of Medicine, Osaka, Japan; 13 Department of Epidemiology, Radiation Effects Research Foundation, Hiroshima, Japan; 14 Department of Public Health, Hokkaido University Graduate School of Medicine, Sapporo, Japan

Correction to: *Scientific Reports* https://doi.org/10.1038/s41598-023-30467-5, published online 01 March 2023

The original version of the Article contained errors in the Consortia author list of The Research Group for the Development and Evaluation of Cancer Prevention Strategies in Japan, where author name, Shiori Tanaka, was duplicated in the main author list.

The original Article has been corrected.

