# Supplementary material for: Response to antiviral therapy for chronic hepatitis C and risk of hepatocellular carcinoma occurrence in Japan: a systematic review and meta-analysis of observational studies
Source: Sci Rep. 2023 Mar 1;13:3445. doi: 10.1038/s41598-023-30467-5 (PMC9977913; doi:10.1038/s41598-023-30467-5)
Supplement: Supplementary file 1 — Supplementary Information. [file 41598_2023_30467_MOESM1_ESM.docx]

**Supplementary file**

**Response to antiviral therapy for chronic hepatitis C and risk of hepatocellular carcinoma occurrence in Japan: a systematic review and meta-analysis of observational studies**

Yoko Yamagiwa, Keitaro Tanaka, Keitaro Matsuo, Keiko Wada, Yingsong Lin, Yumi Sugawara, Tetsuya Mizoue, Norie Sawada, Hidemi Takimoto, Hidemi Ito, Tetsuhisa Kitamura, Ritsu Sakata, Takashi Kimura, Shiori Tanaka, Manami Inoue, for the Research Group for the Development and Evaluation of Cancer Prevention Strategies in Japan

**Supplementary Table 1. Subgroup analyses of pooled estimates of the crude incidence rate (per 100 person-years) and incidence rate ratio of HCC in patients treated with antiviral therapy (SVR *vs*. non-SVR)**

|  | Incidence rate (per 100 person-years)  pooled estimate (95% CI) | | Incidence rate ratio  pooled estimate (95% CI) |
| --- | --- | --- | --- |
|  | SVR | non-SVR |  |
| Study design |  |  |  |
| Retrospective (n = 22) | 0.35 (0.31-0.41) | 1.66 (1.56-1.76) | 0.21 (0.18-0.25) |
| Prospective (n = 3) | 0.51 (0.37-0.70) | 2.07 (1.77-2.44) | 0.25 (0.17-0.35) |
| Institution |  |  |  |
| Single (n = 15) | 0.31 (0.25-0.39) | 1.79 (1.65-1.94) | 0.18 (0.14-0.22) |
| Multi (n = 10) | 0.43 (0.36-0.52) | 1.62 (1.50-1.75) | 0.27 (0.22-0.32) |
| Last year of study period |  |  |  |
| Before 2000 (n = 12) | 0.30 (0.19-0.45) | 1.36 (1.19-1.57) | 0.22 (0.14-0.34) |
| 2000 and later (n = 12) | 0.38 (0.33-0.44) | 1.78 (1.67-1.89) | 0.22 (0.19-0.25) |
| Follow-up duration |  |  |  |
| Less than 5 yrs (n = 13) | 0.40 (0.32-0.50) | 1.74 (1.58-1.93) | 0.23 (0.18-0.29) |
| 5 yrs or longer (n = 11) | 0.36 (0.31-0.43) | 1.68 (1.57-1.80) | 0.22 (0.18-0.26) |
| Sample size |  |  |  |
| Less than 1000 (n = 15) | 0.34 (0.21-0.53) | 1.80 (1.55-2.08) | 0.19 (0.12-0.30) |
| 1000 or more (n = 10) | 0.38 (0.33-0.44) | 1.68 (1.58-1.79) | 0.22 (0.19-0.26) |
| Age |  |  |  |
| Less than 55 yrs (n = 13) | 0.32 (0.26-0.40) | 1.59 (1.47-1.72) | 0.20 (0.16-0.25) |
| 55 yrs or older (n = 11) | 0.42 (0.35-0.50) | 1.82 (1.68-1.97) | 0.23 (0.19-0.28) |

HCC: hepatocellular carcinoma; SVR: sustained virologic response; CI: confidence interval.

**Supplementary Table 2. Subgroup analyses of pooled estimates of the hazard ratio of HCC in patients treated with antiviral therapy (SVR *vs*. non-SVR)**

|  | HR (95% CI) |
| --- | --- |
| Study design |  |
| Retrospective (n = 6) | 0.22 (0.17-0.29) |
| Prospective (n = 1) |  |
| Institution |  |
| Single (n = 3) | 0.19 (0.12-0.29) |
| Multi (n = 4) | 0.31 (0.21-0.46) |
| Last year of study period |  |
| Before 2000 (n = 0) |  |
| 2000 and later (n = 7) | 0.24 (0.18-0.33) |
| Follow-up duration |  |
| Less than 5 yrs (n = 5) | 0.26 (0.16-0.40) |
| 5 yrs or longer (n = 2) | 0.25 (0.14-0.46) |
| Sample size |  |
| Less than 1000 (n = 3) | 0.17 (0.06-0.46) |
| 1000 or more (n = 4) | 0.26 (0.18-0.36) |
| Mean/median age |  |
| Less than 55 yrs (n = 2) | 0.21 (0.15-0.28) |
| 55 yrs or older (n = 5) | 0.32 (0.20-0.50) |

HCC: hepatocellular carcinoma; SVR: sustained virologic response; CI: confidence interval.

**Supplementary Fig 1. Contour-enhanced funnel plot evaluating the publication bias of 8 studies used to determine the pooled estimate of the hazard ratio of HCC incidence adjusted for potential covariates in patients treated with antiviral therapy (SVR *vs.* non-SVR)**

**
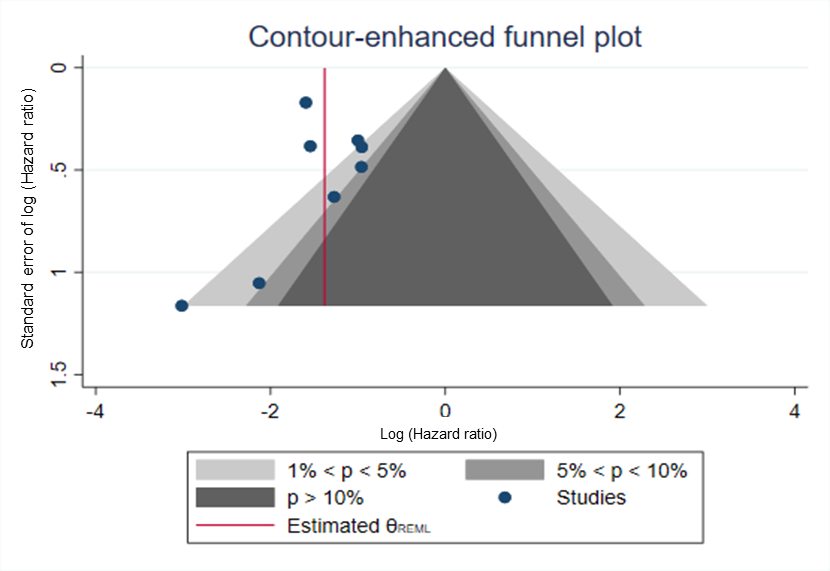
**

HCC: hepatocellular carcinoma; SVR: sustained virologic response; CI: confidence interval.
